# Effect of Extreme Ultraviolet (EUV) Radiation and EUV Induced, N_2_ and O_2_ Based Plasmas on a PEEK Surface’s Physico-Chemical Properties and MG63 Cell Adhesion

**DOI:** 10.3390/ijms22168455

**Published:** 2021-08-06

**Authors:** Joanna Czwartos, Bogusław Budner, Andrzej Bartnik, Przemysław Wachulak, Beata A. Butruk-Raszeja, Adam Lech, Tomasz Ciach, Henryk Fiedorowicz

**Affiliations:** 1 Institute of Optoelectronics, Military University of Technology, 2 Kaliskiego St., 00-908 Warsaw, Poland; boguslaw.budner@wat.edu.pl (B.B.); andrzej.bartnik@wat.edu.pl (A.B.); przemyslaw.wachulak@wat.edu.pl (P.W.); adam.lech@wat.edu.pl (A.L.); henryk.fiedorowicz@wat.edu.pl (H.F.); 2 Faculty of Chemical and Process Engineering, Warsaw University of Technology, Waryńskiego 1, 00-645 Warsaw, Poland; Beata.Raszeja@pw.edu.pl (B.A.B.-R.); tomasz.ciach@pw.edu.pl (T.C.); 3 Centre for Advanced Materials and Technologies CEZAMAT, Warsaw University of Technology, Poleczki 19, 02-822 Warsaw, Poland

**Keywords:** PEEK, extreme ultraviolet (EUV), nitrogen and oxygen low-temperature plasma, surface modification, surface chemistry, XPS, AFM, cell adhesion, human osteoblast-like MG63 cells

## Abstract

Polyetheretherketone (PEEK), due to its excellent mechanical and physico-chemical parameters, is an attractive substitute for hard tissues in orthopedic applications. However, PEEK is hydrophobic and lacks surface-active functional groups promoting cell adhesion. Therefore, the PEEK surface must be modified in order to improve its cytocompatibility. In this work, extreme ultraviolet (EUV) radiation and two low-temperature, EUV induced, oxygen and nitrogen plasmas were used for surface modification of polyetheretherketone. Polymer samples were irradiated with 100, 150, and 200 pulses at a 10 Hz repetition rate. The physical and chemical properties of EUV and plasma modified PEEK surfaces, such as changes of the surface topography, chemical composition, and wettability, were examined using atomic force microscopy (AFM), scanning electron microscopy (SEM), X-ray photoelectron spectroscopy (XPS), and goniometry. The human osteoblast-like MG63 cells were used for the analysis of cell viability and cell adhesion on all modified PEEK surfaces. EUV radiation and two types of plasma treatment led to significant changes in surface topography of PEEK, increasing surface roughness and formation of conical structures. Additionally, significant changes in the chemical composition were found and were manifested with the appearance of new functional groups, incorporation of nitrogen atoms up to ~12.3 at.% (when modified in the presence of nitrogen), and doubling the oxygen content up to ~25.7 at.% (when modified in the presence of oxygen), compared to non-modified PEEK. All chemically and physically changed surfaces demonstrated cyto-compatible and non-cytotoxic properties, an enhancement of MG63 cell adhesion was also observed.

## 1. Introduction

PEEK is a high-temperature, thermoplastic polymer, with mechanical and physico-chemical properties which make it an attractive alternative for metal implants commonly used in reconstructive surgery (maxillofacial surgery, spine surgery, orthopedic surgery, etc.) [1,2]. PEEK is biocompatible, however—as most of the organic polymers—is also bioinert due to the low surface hydrophilicity and lack of polar surface chemical groups what, in turn, significantly limits proteins adsorption and cell adhesion [3,4,5,6,7]. As a consequence, when introduced into the human body, PEEK limits osseointegration (e.g., by limiting the formation of bone at the fracture site). In order to improve the polymer-tissue integration, PEEK’s surface, in particular, its chemical surface composition and topography, must be modified to raise its ability of cell attachment. Many techniques have been used to improve PEEK surface bioactivity, including chemical modification [8,9], HA deposition [10,11], laser processing [12,13], and one of the most popular and important processing technologies: modification using low-temperature plasma [14,15]. Exposure of polymers to plasma induces various changes on their surfaces such as surface ablation, crosslinking, etching, chemical changes such as the formation of polar functional groups or incorporation of atoms, topography change manifested by a change in surface roughness, etc. As a consequence, surfaces that have good wettability and promote cell adhesion and proliferation can be obtained. The effect of plasma impact on PEEK’s physico-chemical properties and its bioactivity have been investigated by many authors. For example, Liu et al. [16] observed that PEEK’s surface treated with Ar, N_2_, and the mixture of Ar + N_2_ plasma induced strong chemical changes (i.e., incorporation of nitrogen atoms) and increase of roughness (formation of nano-patterns) resulting in a significantly increased wettability. Also, it turned out that all three modification variants used (three types of plasma) improved osteogenic activity as well as the antibacterial performance of PEEK. Specifically, N_2_ plasma treatment increased osteogenic activity the most, whereas Ar and Ar + N_2_ plasma treatment impacted the antibacterial performance the most. Fu et al. [17] modified the PEEK surface using three types of plasmas: hydrogen, oxygen, and H_2_/O_2_ mixture. They observed that all PEEK surfaces modified this way presented improved hydrophilicity, crystallinity, and micro-hardness properties. This led to the enhancement of the adhesion and proliferation rate of human osteoblasts seeded on the PEEK surface. Other studies have shown that modifications of PEEK surfaces using low-temperature plasmas can significantly improve its adhesive properties which are very important when applying organic or non-organic coatings [18,19,20,21,22].

In this study, an alternative technique of surface modification was used. It was based on simultaneous exposure to intense nanosecond pulses of extreme ultraviolet (EUV) and low-temperature plasmas, induced by the EUV radiation near the sample surface. Plasmas were created in gases injected in the vicinity of the irradiated surface, synchronously with the EUV pulses. Two molecular gases were used for the production of such plasmas: oxygen and nitrogen. Plasmas were created employing an EUV irradiation system designed in our laboratory especially for this purpose [23]. The advantages and differences of this technique, compared to standard techniques used for modification of polymers, have been described in our previous papers [4,24]. Exposure of the surfaces of the polymer to the EUV radiation leads to surface ablation, topography, and chemical composition alterations. Moreover, additional usage of the low-temperature plasma induced by the EUV beam in a gas (nitrogen or oxygen), injected into the vicinity of the irradiated polymer surface causes the formation of new chemical groups on the polymer surface and incorporation of nitrogen or oxygen atoms. In this paper, the detailed chemical analysis of modified PEEK foils carried out based on high-resolution XPS spectra analysis is presented. Changes in surface topography were examined using atomic force microscopy (AFM) and scanning electron microscopy (SEM). Water contact angle measurement was employed to evaluate the wettability of the EUV and plasma-treated PEEK samples. Finally, the influence of physico-chemical changes of modified PEEK samples on the behavior of human osteoblast-like MG63 cells was evaluated.

## 2. Results and Discussion

### 2.1. Changes in Surface Morphology of the PEEK Surfaces

**Figure 1:**
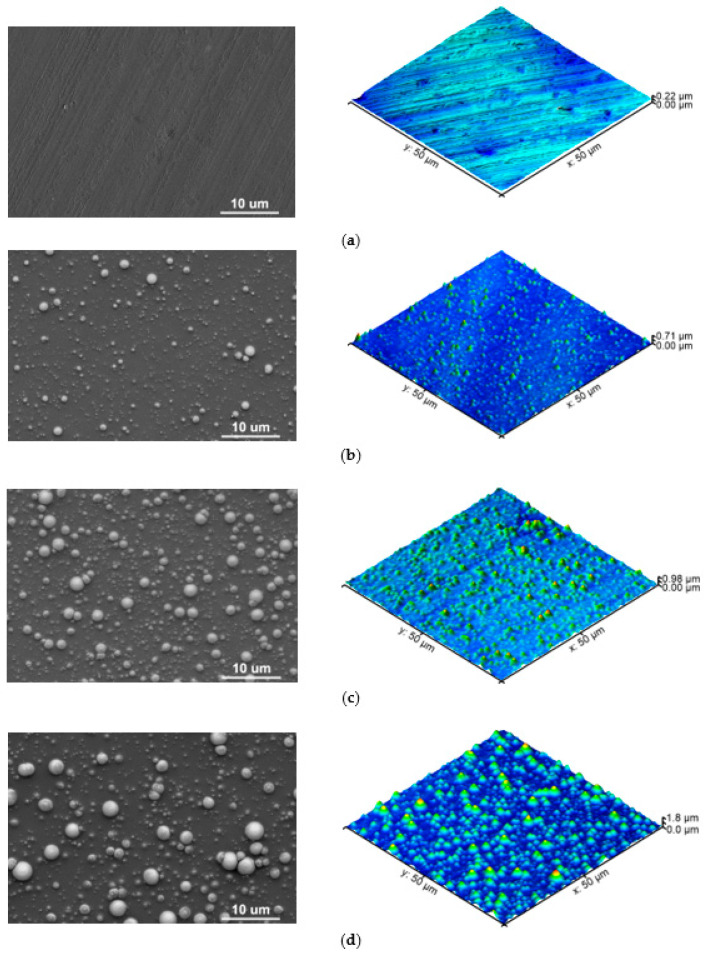
SEM images compared with the AFM topographies of (**a**) PEEK pristine, (**b**) PEEK treated with 100 EUV pulses, (**c**) PEEK treated with 150 EUV pulses, and (**d**) PEEK treated with 200 EUV pulses.

Interaction of nanosecond EUV pulses with polymers induces changes in the morphology of their surfaces in form of various micro- and nano-patterns. The character of these changes depends not only on the physico-chemical structure of a polymer, such as the chemical structure of a mer unit, sensitivity to radiation, degree of crystallinity, the temperature of phase transitions, or ablation thresholds, but also on the number of pulses used, pulse length or fluence. In this experiment, the effect of simultaneous interaction of 100, 150, and 200 EUV radiation pulses and oxygen or nitrogen plasma on the PEEK surface morphology was analyzed using atomic force microscopy (AFM) and electron scanning microscopy (SEM). Figure 1 presents AFM topographies of the PEEK surfaces in 3D with a scan size of 50 µm × 50 µm as well as SEM scans of these surfaces, with the same scale. As it did not matter, for the patterns formed on the PEEK surface, if the modification was performed in the presence of nitrogen or oxygen (in other words, structures formed on the PEEK surface were the same in both cases in terms of their heights, shapes, etc.), this paper discusses only the results of the PEEK surface modifications using EUV radiation and nitrogen plasma. It is worth noting that the formation of the structures on the irradiated polymers depends mainly on the EUV radiation, whereas adding gas leads to chemical changes on the surface through the incorporation of atoms of the gas ionized with EUV radiation.

In order to inspect the changes on the polymer’s surface resulted from EUV radiation, first, the PEEK pristine surface was examined. Figure 1a presents the topography of PEEK pristine.

**Figure 2:**
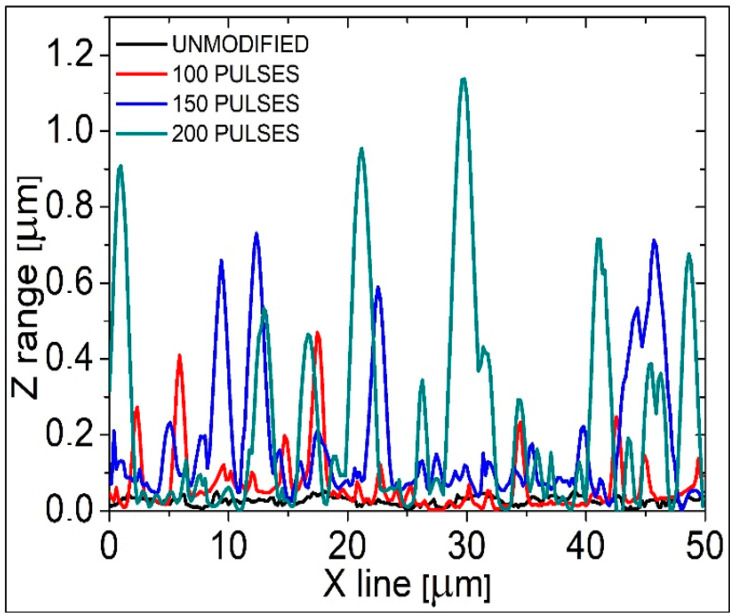
Cross-section profiles obtained from the AFM topographies along the selected line for unmodified PEEK and PEEK modified with 100, 150, and 200 EUV pulses, respectively.

As can be seen, the surface of non-modified PEEK is relatively smooth, and patterns on this surface, being the natural irregularities running in one direction, were most probably formed in the process of production, through the contact with roller or press. The heights of these structures, measured through the cross-section analysis (along a single selected line), do not exceed 50 nm (Figure 2), and the surface roughness (root mean square–RMS) was 14.6 nm ± 2.5 nm.

Figure 1b shows the PEEK surface irradiated with 100 EUV pulses in the presence of nitrogen plasma. Modified polymer’s surface changed significantly comparing to the PEEK pristine and nano- and micro-conical structures were formed on the whole surface. The highest cones reach up to ~500 nm and the diameter of their bases is ~2 µm. The majority of the smaller cones are 70–120 nm in height—Figure 2 (red line). The RMS roughness of the surface is almost three times higher compared to the non-modified sample and amounts to 48.4 nm ± 3.9 nm. The density of the conical structures on the PEEK surface sample modified with 150 EUV pulses is higher compared to the one modified with 100 EUV pulses. They are also higher, up to ~700 nm (Figure 2—blue line). The cones’ bases are bigger, too, and their diameters are up to ~3 µm, and this sample’s roughness is 162.0 nm ± 9.9 nm. The last case, which is the PEEK surface modified with 200 EUV pulses is the sample on which the highest conical structures (exceeding 1 µm) were observed. The surface roughness was the highest, too, and amounted to 253.3 nm ± 21.1 nm. In conclusion, the more pulses used, the higher conical structures were present on the surface of the samples and their surface roughness was increased as well.

Conical structures were observed by many other researchers who irradiated various organic polymers such as, PI, PET, PEN, PC, N6, etc. using UV lasers [25,26,27,28,29]. Our research group also observed similar conical structures in previous experiments in which polymers such as PET, PMMA, FEP, and PTFE, were exposed to EUV pulses [30,31,32,33]. The mechanism of the formation of these structures is not yet clearly understood. However, the process of the formation of the conical structures has often been explained by a shielding effect of some surface or volume impurities having a higher ablation threshold than the polymer [25,26,27,34].

### 2.2. Chemical Analysis of Non-Modified and Modified PEEK Surfaces

**Figure 3:**
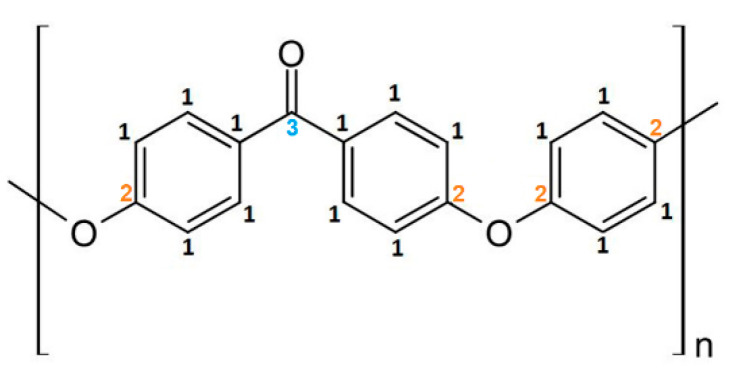
Chemical structure of PEEK’s mer.

The chemical composition analysis of the PEEK samples modified in the presence of nitrogen and oxygen started from the analysis of the non-modified PEEK being the reference material. The non-modified PEEK‘s chemical structure is presented in Figure 3.

**Figure 4:**
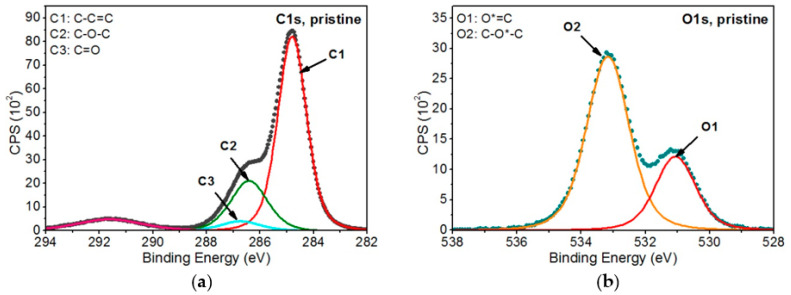
XPS spectra of pristine PEEK: (**a**) C1s band and (**b**) O1s band [35].

When analyzing the chemical structure of the non-modified PEEK material, three chemical forms in C1s band and two chemical forms in O1s band were specified (Figure 4):

- C-C=C (C1)—at 284.8 eV (FWHM 1.3 eV) represents the structure in carbon rings (carbon atoms marked as 1 in Figure 3).
- C*-O-C* (C2)—at 286.4 eV (FWHM 1.6–1.7 eV)—carbon atoms marked with 2 on Figure 3.
- C*=O (C3)—at 286.7 eV (FWHM 1.7–1.8 eV)—carbon atoms marked with 3 on Figure 3.
- O*=C (O1)—at 531.1 eV (FWHM 1.5 eV).
- C-O*-C (O2)—at 533.2 eV (FWHM 1.6 eV).

**Figure 5:**
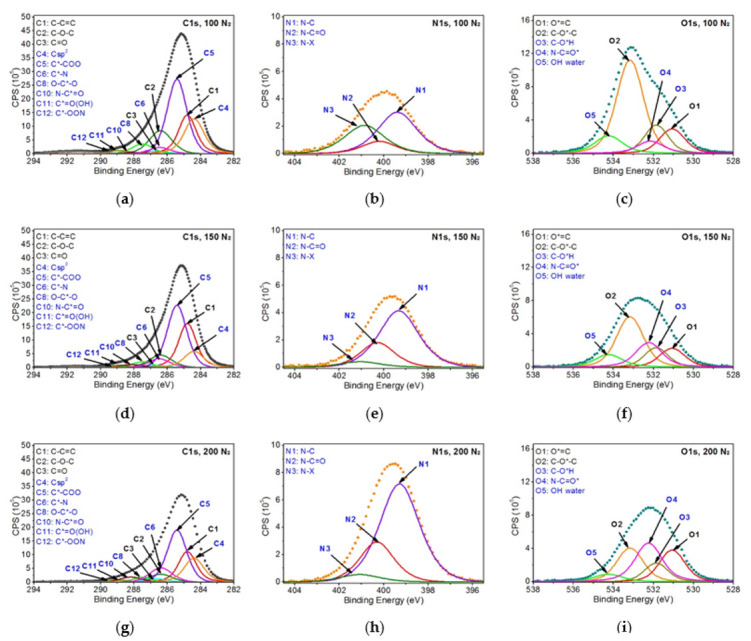
XPS spectra of PEEK modified with EUV radiation and nitrogen plasma: (**a**) C1s band—100 EUV pulses, (**b**) O1s band—100 EUV pulses, (**c**) N1s band—100 EUV pulses, (**d**) C1s band—150 EUV pulses, (**e**) O1s band—150 EUV pulses, (**f**) N1s band—150 EUV pulses, (**g**) C1s band—200 EUV pulses, (**h**) O1s band—200 EUV pulses, and (**i**) N1s band—200 pulses.

**Figure 6:**
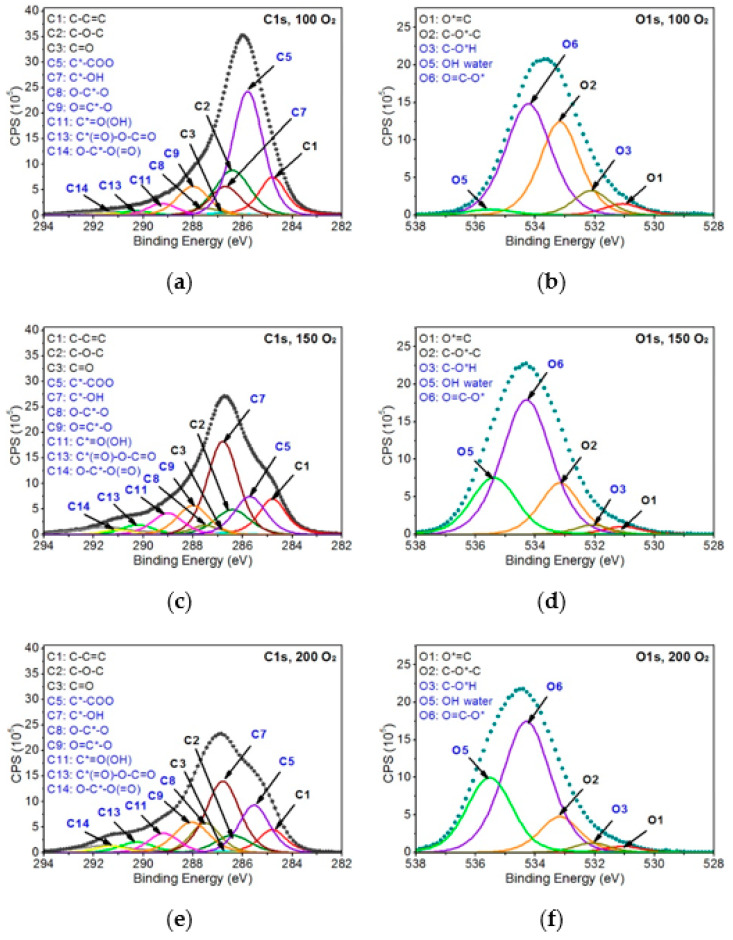
XPS spectra of PEEK modified with EUV radiation and oxygen plasma: (**a**) C1s band—100 EUV pulses, (**b**) O1s band—100 EUV pulses, (**c**) C1s band—150 EUV pulses, (**d**) O1s band—150 EUV pulses, (**e**) C1s band—200 EUV pulses, and (**f**) O1s band—200 EUV pulses.

Further analysis of the modified PEEK samples was carried out based on the model of non-modified PEEK. The peaks present in the reference material were introduced to the C1s and O1s bands of the modified samples and, additionally, the peaks correlated with expected changes in the chemical structure on the surface of the modified samples were introduced. All the peaks were modeled, and their parameters and their atomic percentages are given in Table 1. Figure 5 and Figure 6 show all analyzed XPS spectra for PEEK modified with EUV radiation and nitrogen plasma and for PEEK modified with EUV radiation and oxygen plasma.

As can be seen in Table 1, the reference material peaks and 13 peaks, being the result of changes in the chemical structure, were specified for the PEEK samples modified in the presence of nitrogen. As for the PEEK modified in the presence of oxygen, 10 additional peaks, being the result of modification, were introduced. All these peaks were assigned to the following functional groups:

- C sp2 (C4)—at 284.4 eV (FWHM 1.4 eV)—as this peak is shifted to lower binding energies than the C1 peak associated with the carbon ring of the non-modified material, it was considered that it is highly probable that the C4 peak represents carbon rings in which hydrogen and oxygen atoms have been detached. Therefore, the structure formed on the modified surface obtained the characteristics of a graphite material, which is described as carbon in the form of sp2.
- C*-COO (C5)—at 285.4–285. eV (FWHM 1.4–1.5 eV)—this peak is related to secondary chemical shifts. It is most probably a result of the break of the bonds between atoms marked as 3 and 2 (Figure 3), which linked adjacent carbon rings.
- C*-N (C6)—at 286.4–286.5 eV (FWHM 1.5–1.6 eV)—this peak is related to the incorporation of nitrogen atoms into the PEEK structure being the result of break of the bonds of the carbon marked as 2 or 3 (Figure 3) and introduction of nitrogen atoms in that place.
- C*-OH (C7)—at 286.7–286.9 eV (FWHM 1.5–1.6 eV)—this peak is related to the OH groups formed on the surface of modified PEEK, connected with carbon atoms.
- O-C*-O (C8)—at 287.5–287.6 eV (FWHM 1.3–1.6 eV)—the peak is most likely the result of breaking the bond between the carbon marked as 3 (Figure 3) and the carbon ring. The empty bond is then filled with an oxygen atom.
- O=C*-O (C9)—at 288.0–288.1 eV (FWHM 1.5–1.7 eV)—according to the literature [36], this peak’s position corresponds the most to the carbon atom structure presented bonded with two oxygen atoms. A structure like this most probably forms as a result of breaking bonds between carbon atoms, marked with 3 and 1, and attaching at that place an additional oxygen atom.
- N-C*=O (C10)—at 288.1–288.2 eV (FWHM 1.6–1.7 eV)—the peak most probably formed as a result of carbon bond breaking (carbon marked as 3—Figure 3) and filling it with a nitrogen atom.
- C*=O(OH) (C11)—at 288.9–289.2 eV (FWHM 1.4–1.7 eV)—the peak most probably formed as a result of breaking the bond between the atom marked as 3 (Figure 3) and the carbon ring and then attaching the OH group to the carbon atom marked as 3.
- C*-OON (C12)—at 289.4–289.5 eV (FWHM 1.3–1.5 eV)—due to a huge chemical shift of this peak relative to the C1 peak, it was assumed that this structure most probably contains two oxygen atoms and a single nitrogen atom. The proof for the presence of this peak is a huge change of the shape of the C1s’ envelope around the binding energy values of 289.3–289.5 eV. The presence of the peak is also confirmed by the incorporation of nitrogen atoms.
- C* -(=O)-O-C=O (C13)—at 290.2–290.3 eV (FWHM 1.5–1.6 eV)—as the peak is strongly shifted towards higher binding energy, its position most probably represents this structure [36].
- O-C*(=O)-O (C14)—at 291.0–291.3 eV (FWHM 1.4–1.5 eV)—this peak is the most shifted towards higher binding energy. According to the literature, such position of this peak indicates that the carbon atom must be bonded with three oxygen atoms and must be double-bonded with at least one of them [36].
- N*-C (N1)—at 399.3–399.4 eV (FWHM 2.1 eV)—this peak corresponds to C6.
- N*-C=O (N2)—at 400.2–400.3 eV (FWHM 1.8 eV)—this peak corresponds to C10.
- N*-x (N3)—at 400.9–401.1 eV (FWHM 2.1 eV)—this peak is difficult to interpret in undisputable manner. Referring to the examinations of the reference polymers which contain nitrogen atoms (nylon, PU, Kapton) and confronting with literature data, one can assume these are nitrogen atoms bonded with carbon atoms forming chemical bonds with at least two oxygen atoms.
- C-O*-H (O3)—at ~531.9–532.1 eV (FWHM 1.4 eV)—this peak corresponds with the C*-OH group (C7). The OH group can be formed as a result of the oxygen plasma modification as well as due to filling empty bonds in the material structure after taking the sample out from the vacuum chamber (the reaction of water vapor from the atmosphere with the reactive PEEK surface).
- N-C=O* (O4)—at 532.2 eV (FWHM 1.5–1.6 eV)—this peak corresponds to C10.
- OH (water) (O5)—at 534.2–535.5 eV (FWHM 1.6–1.8 eV)—this peak most probably comes from OH groups of the water adsorbed on the surface of the sample after it has been taken from the vacuum.
- O=C-O* (O6)—at 534.2–534.3 eV (FWHM 1.8–1.9 eV)—this peak position corresponds to the oxygen atoms single-bonded with carbon atom which is double bonded with another oxygen atom. A structure like this can be formed as a result of breaking one of the carbon atoms bonds, marked as 3, in the PEEK chemical structure (Figure 3) and placing an oxygen atom in this place.

Table 1 and Table 2 show clear relation between the percentage content of carbon, nitrogen, and oxygen atoms, functional groups described above, and conditions in which modifications were done. The modification in the presence of oxygen resulted in a significant increase in oxygen percentage content on the samples’ surfaces compared to the reference material. The increase of oxygen percentage content from 13.9 at.% for the non-modified PEEK up to 20.6 at.% for PEEK modified with 100 EUV pulses (PEEK-O_2_-100), and up to 25.7 at.% for PEEK modified with 200 pulses (PEEK-O_2_-200) was observed. This means the percentage content of oxygen for the latter sample doubled compared to the non-modified sample. An increase in the content of oxygen results in a decrease in the percentage content of carbon. As far as the modification in the presence of nitrogen is concerned, incorporation of nitrogen into the PEEK material structure was observed, as expected. There is an explicit relation between the number of pulses and the percentage content of nitrogen. The percentage content of nitrogen ranges from 5.5 at.% for PEEK modified with 100 EUV pulses (PEEK-N_2_-100) to 7.4 at.% for PEEK modified with 150 EUV pulses (PEEK-N_2_-150), and to 12.3 at.% for PEEK modified with 200 EUV pulses (PEEK-N_2_-200). Doubling the number of pulses leads to more than a double increase in the percentage content of nitrogen. As the modification was performed in non-oxygen conditions, a slight decrease in the percentage content of oxygen compared to reference material was also observed, to 10.2 at.% for PEEK-N_2_-100, 9.2 at.% for PEEK-N_2_-150, and 9.4 at.% for PEEK-N_2_-200. For samples modified with 150 and more pulses, the decrease in the percentage content of the oxygen is random rather than proportional to the number of pulses.

Referring to Table 1 and the percentage content of the specific functional groups it lists, it is observed that, first of all, the percentage content of C1–C3 and O1–O2 peaks coming from non-modified PEEK significantly decreases in all modified samples. It follows the expectations as modifications performed using EUV radiation and N_2_ or O_2_ low-temperature plasma leads to the bond breaking in the chemical structure of the PEEK and the formation of the new functional groups on its surface.

The percentage content of C1 peak is 62.3 at.% in non-modified PEEK, ranges between 15.7 at.% and 22.2 at.% in the PEEK modified in the presence of nitrogen and amounts from 7.2 at.% to 10.7 at.% in the PEEK modified in the presence of oxygen. The C2 peak percentage content lowers from 19.9 at.% in the pristine PEEK to 4.9–11.1 at.% in PEEK modified with nitrogen and to 6.3–14.9 at.% in PEEK modified with oxygen. The percentage content of C3 decreases from 4 at.% in non-modified PEEK to 1.3–2.2 at.% in PEEK modified with nitrogen and to 0.5–0.9 at.% when modified with oxygen. In all these cases, a significant decrease in the percentage content of these peaks is therefore observed. It can also be noticed that the modifications in the presence of oxygen are generally more aggressive and make the percentage content of PEEK pristine peaks significantly lower than in the case of modifications using nitrogen. However, in some cases, it can be noticed that the percentage content of the C1–C3 peaks becomes random and loses its correlation with the number of pulses. The same applies to the O1s band (peaks O1–O2), as the percentage content of peaks also decreases comparing to the pristine PEEK after the modification has been done. The modification leads to the transformation of these reference material’s functional groups into different chemical structures or to detaching oxygen atoms from the polymer structure and removing them from the material. The process of removing some of the atoms (oxygen atoms and specifically hydrogen atoms), is observed mainly during the modification with nitrogen resulting in the formation of the C4 peak, described as Csp^2^. The percentage content of the C4 peak is quite high and ranges from 9.2 at.% to 15.9 at.%. The modification leads also to the formation of numerous new chemical groups as a result of breaking bonds between atoms in the PEEK structure and incorporating oxygen or nitrogen atoms.

The percentage content of the new functional groups depends on modification conditions and ranges from 0.2 at.% up to 36.3 at.%. These are mainly chemical structures formed by attaching one or more oxygen or nitrogen atoms to carbon atoms in the original PEEK material. It is the C*-COO (C5) peak corresponding to the secondary chemical shift that has the highest percentage content in all the samples. Most probably, this specific peak will be formed on the surface as the outcome of the fragmentation of polymer chains and introduction of oxygen atoms into their empty ends. The percentage content of this peak ranges from 26.7 at.% to 32.1 at.% for the PEEK modified with nitrogen (which can be the result of fragmentation of polymer chains at the atom marked as 3 (Figure 3). In the PEEK modified with oxygen, the percentage content of this peak ranges from 12.2 at.% to 36.3 at.% which is the result of polymer chains fragmentation and filling empty bonds with oxygen atoms. The wider range of this group’s percentage content in the samples modified in the presence of oxygen indicates that not only do polymer chains become fragmented but oxygen atoms from plasma become incorporated as well. As a result, the percentage content of C5 in the samples modified with oxygen is lower and the formation of other functional groups, i.e., C7–C9, C11, C13, C14, and O6 is observed, and their total percentage content is relatively high. The OH group (C7) dominates over the others and its percentage content amounts between 8.4 at.% and 29.6 at.%. The percentage content of the other groups in which more than one oxygen atom is attached in various configurations to a carbon atom, i.e., C8, C9, C11, C13, C14, is significantly lower, from 0.2 at.% to 9.2 at.% at maximum.

As mentioned before, some of the peaks corresponding to the chemical groups formed as a result of attaching the oxygen atoms to carbon atoms (C7, C9, C13, C14, and O6) do not appear during modification in the presence of nitrogen. Instead, three characteristic peaks, i.e., C6, C10, and C12, corresponding with N1–N3 peaks from the N1s band, are formed in the C1s band; also another characteristic peak, O4, is formed in the O1s band. The percentage content of peaks C6, C10, and C12 is low, from 0 to 8.6 at.%. It is the peak C6 corresponding with the group C*-N that dominates, and its percentage content increases from 2.8 at.% to 8.6 at.% as the number of pulses rises. The percentage content of the rest of the peaks, i.e., C10 and C12, also increases with the rise of the number of pulses, however, this increase is much smaller.

### 2.3. Wettability

**Figure 7:**
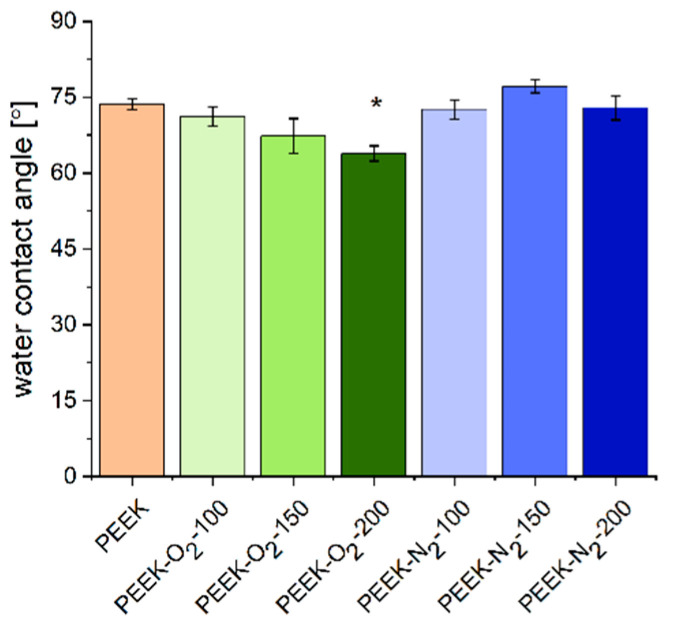
Water contact angle (WCA) measured for analyzed surfaces: non-modified and modified PEEK. MV ± SD, *n* = 9, * *p* < 0.05 vs. control (PEEK).

The water contact angle (WCA) measurement results are shown in Figure 7. The WCA of control material (PEEK) equaled to 74 ± 3°, which is closed to values reported by other authors for untreated PEEK [16,17,37,38]. The modification in the presence of oxygen slightly increased the wettability of the PEEK surface, the contact angle decreased with the increase of the number of EUV pulses and amounted to 71 ± 6°, 67 ± 10° and 64 ± 5° for PEEK-O_2_-100, PEEK-O_2_-150 and PEEK-O_2_-200, respectively. The value measured for PEEK-O_2_-200 was significantly lower (*p* < 0.05) compared to the control. Other authors also reported a decrease in WCA values for PEEK treated with plasma in the presence of oxygen [17]. PEEK modified using nitrogen gas did not show significant differences in WCA values compared to the control material. The WCA values were respectively: 73 ± 6°, 77 ± 4° and 73 ± 7° for PEEK-N_2_-100, PEEK-N_2_-150, and PEEK-N_2_-200. This may lead to the observation, that oxygen modification leads to a more polar behavior, hence better water wet-able surface. Data presented in the literature also show that the modification of PEEK under a nitrogen atmosphere mostly leads to an increase in surface hydrophilicity [16,38].

In our study, the modification process conducted in the presence of oxygen resulted in the formation of hydrophilic C-OH groups (Table 1). The number of newly created groups was the lowest for the material modified with the smallest number of pulses, PEEK-O_2_-100 (8.4 at.%). For larger numbers of pulses, a higher percentage of C-OH groups was obtained, with the highest number for PEEK-O_2_-150 (29.6 at.%) and slightly lower for PEEK-O_2_-200 (25 at.%). A similar relationship is observed between the number of pulses and the WCA value—as the number of pulses increases, the hydrophilicity of the surface increases. It is worth noting that the C-OH groups were not present in the control material. Also, the presence of that group was not demonstrated in materials modified in the presence of nitrogen gas. Similar results were obtained in our previous studies on modifying other polymers (PTFE, PLLA, PVDF) [39]. In the study, we also observed lower WCA values for surfaces modified in an oxygen atmosphere compared to those modified in a nitrogen atmosphere.

### 2.4. Cell Adhesion and Viability

**Figure 8:**
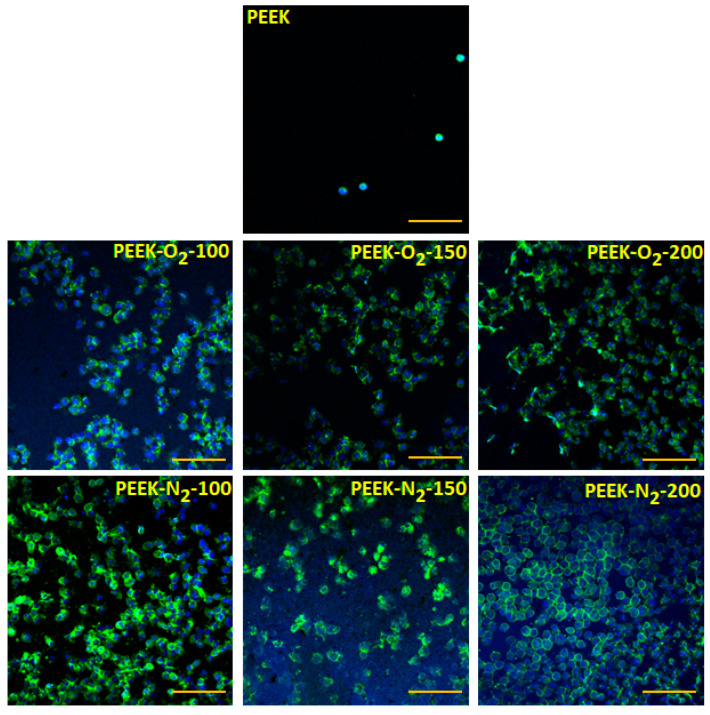
MG63 adhesion to the analyzed materials after 12 h of culture. Scale bar: 100 μm. Blue staining—nucleus (DAPI), green staining—actin (AlexaPhalloidin 488).

**Figure 9:**
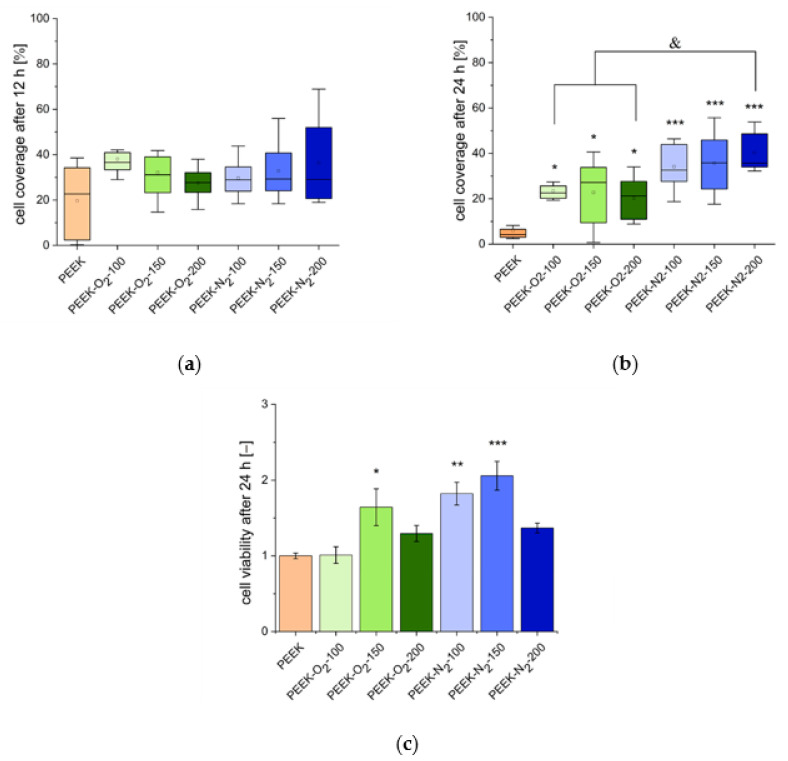
MG63 cell coverage after 12 (**a**) and 24 h (**b**) of culture and cell viability after 24 h of culture (**c**). MV ± SD, *n* = 9, * *p* < 0.05, ** *p* < 0.01, *** *p* < 0.005 vs. control (PEEK), & *p* < 0.05.

Microscopic analysis of human osteoblast-like cells (MG63) growing on the tested surfaces after 12 h of culture (Figure 8) showed an increased number of cells on all the modified PEEK surfaces as compared to the control surface (PEEK). The cells showed normal morphology and were homogeneously distributed over the entire analyzed area. The percentage of the cell-occupied area (cell coverage, Figure 9a) was 20 ± 16% for control material (PEEK). For modified materials, it increased by at least 10 percentage points and amounted to: 38 ± 7% (PEEK-O_2_-100), 32 ± 12% (PEEK-O_2_-150), 28 ± 6% (PEEK-O_2_-200), 30 ± 8% (PEEK-N_2_-100), 33 ± 12% (PEEK-N_2_-150), and 36 ± 19% (PEEK-N_2_-200). There was no correlation between the number of cells and the number of pulses used in the modification process. Similarly, no significant differences were observed for PEEK modified with nitrogen versus oxygen.

**Figure 10:**
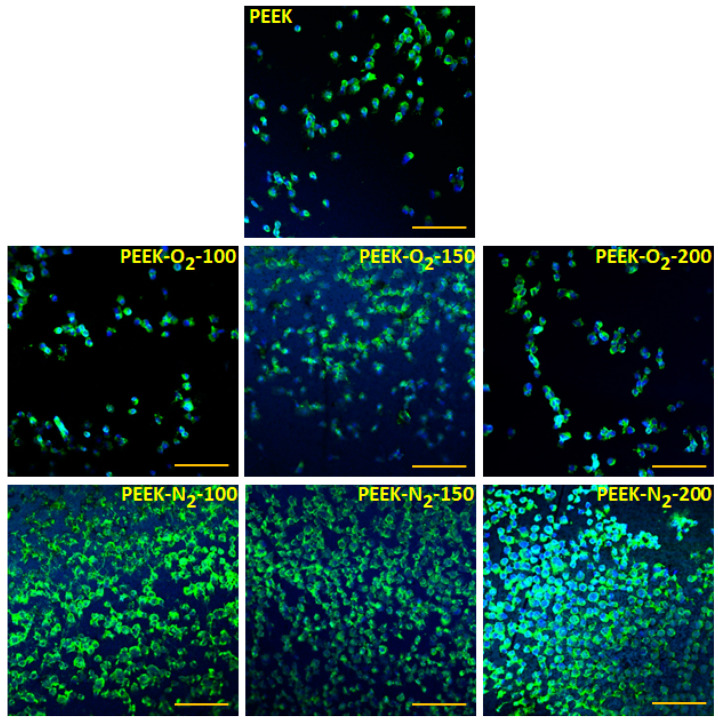
MG63 adhesion to the analyzed materials after 24 h of culture. Scale bar: 100 μm. Blue staining—nucleus (DAPI), green staining—actin (AlexaPhalloidin 488).

A stronger effect of the modification was noticed after 24 h of cell culture (Figure 9b). In the case of the control material (PEEK), the percentage of the area occupied by cells decreased significantly, reaching the value of 6 ± 5%. Microscopic observation revealed the presence of single cells with spherical morphology (Figure 10). The dependence of the number of cells on the type of gas used in the modification process was clearly visible. For all materials modified in the presence of oxygen, the cell coverage was significantly higher compared to the control material (*p* < 0.05). However, this value decreased comparing to the values obtained after 12 h of culture and amounted to: 23 ± 7% (PEEK-O_2_-100), 23 ± 14% (PEEK-O_2_-150), and 20 ± 9% (PEEK-O_2_- 200). In the case of materials modified in a nitrogen atmosphere, the cell coverage values slightly increased compared to the values observed after 12 h of culture and amounted to: 34 ± 9% (PEEK-N_2_-100), 36 ± 13% (PEEK-N_2_-150), and 40 ± 8% (PEEK-N_2_-200). All values were statistically significantly higher than those for the control material (*p* < 0.05). The highest cell coverage was achieved for the surface modified with the highest number of pulses (PEEK-N_2_-200). Also, the cell coverage for PEEK-N_2_-200 was statistically significantly higher (*p* < 0.05) compared to each oxygen-modified surface.

It is worth noting that for most of the analyzed surfaces the observed cell coverage was not uniform—there were areas with a higher or lower number of cells. The cell counts were made for eight photos taken for randomly selected areas. This explains the relatively high SD values obtained for the measurements.

Cell viability performed after 24 h of culture (Figure 9c) revealed that all analyzed surfaces were not cytotoxic. All variants of the modified PEEK surfaces showed a similar or higher viability rate compared to the control surface. In most cases, for materials modified in a nitrogen atmosphere, cell viability was higher compared to the control and to the materials modified in an oxygen atmosphere, which is obviously related to the higher number of cells adhered to these surfaces.

The analysis of the cell adhesion process leads to the conclusion that during the first hours of culturing (time point = 12 h) it is the surface morphology that plays the main role in cell adhesion. For all the modified PEEK materials, increased roughness was observed compared to the control material. This roughness increased with the increasing number of pulses. Additionally, the modification process resulted in the appearance of nano- and micro-conical structures of various heights and sizes (depending on the number of pulses used). Our study showed that there was no relation between the roughness of modified surfaces and the number of cells adhered to those surfaces observed. However, a strong effect of the performed modification process, regardless of the gas used and the number of pulses, on cell adhesion was demonstrated. Therefore, it seems that during the first hours of cell culture the most important factor is the change from a relatively flat surface morphology (control material, RMS = 14.6 nm) to a rough surface containing three-dimensional nano- and micro-objects, regardless of their number and height (RMS above 45 nm).

During longer culturing (time point = 24 h), the relationship between the number of surface-adhered cells and the type of gas used in the modification process was demonstrated. Nitrogen-modified and oxygen-modified surfaces differ in the chemical composition of the surface. The oxygen-modified materials showed higher oxygen content (over 20%) compared to the control material (13.9%) and nitrogen-modified materials (approx. 10%). On the other hand, the materials modified with nitrogen were the only ones that contained nitrogen in the surface composition (over 5%), the percentage of nitrogen increased with the increase in the number of pulses used. It can therefore be concluded that at this stage of the cell culture other factors (apart from surface morphology) begin to matter—it is certainly the chemical composition of the surface and the presence of specific chemical groups.

Many reports are studying the relationship between the surface chemistry of plasma-treated surfaces and cell adhesion. Boesplug et al. [40] investigated the influence of the chemical composition of the plasma-modified PET surface on the adhesion of endothelial cells. Three types of materials were tested: N-functionalized, O-functionalized, and hybrid surfaces (N+O-functionalized). It was shown that both N-rich surfaces and O-rich surfaces increased cell adhesion compared to unmodified PET. This effect was visible after 4 h of adhesion and persisted after 24 h of culture. Surprisingly, hybrid surfaces containing both N and O showed a lower number of surface-adhered cells.

Many studies show that N-rich surfaces, especially aminated surfaces, strongly promote the adhesion of different types of cells. It has also been shown that for a given cell type there is a critical concentration of N, below which the processes of cell adhesion do not occur. Girard-Lauriault et al. [41] observed the adhesion of three types of cells on micropatterned nitrogen-rich polypropylene (PPE). They proved that cells adhered and proliferated only on the N-rich islands, but not elsewhere on the polymer surfaces. The authors suggested the existence of a “critical” value, [N] crit, necessary to induce cell adhesion. In the case of all three tested cell types (cartilage cells, chondrocytes, and macrophages) it was 25% or more. Surfaces with lower values of [N] did not induce cell adhesion [42].

Other works are showing the opposite relationship. Zhang et al. [43] showed that the plasma modification of PET surface in a nitrogen atmosphere (surface containing C-N and C=N groups) reduced osteoblasts proliferation compared to surfaces containing C-O and C=C. Ertel et al. [44] have shown that while an N-containing plasma polymer promotes fibronectin adsorption, it does not initiate cell attachment.

To better understand these unambiguous relationships, it is worth recalling the mechanisms of cell adhesion. It is well described that implanted materials are coated with host proteins during contact with blood or other body fluids, which is called the Vroman effect [45]. Those proteins form an intermediate layer, participating in the processes of anchoring and signal transmission on the cell-environment and cell-cell path. Thus, the composition of the adsorbed layer is a key factor in the cell adhesion processes. The molecular interfacial mechanisms of cell attachment to N-containing polymers have been studied by Steele et al. [46], who studied plasma-modified fluoroethylenopropylene. Results showed that N-containing surfaces enable the adhesion of cells through two possible pathways, by increasing adsorption of fibronectin or vitronectin. Such a dual-mode of cell adhesion support was not found in other, non-N-containing surfaces. Moreover, the N-containing surfaces did not cause conformational changes in the adsorbed proteins, which allowed to maintain the native and active structure.

Studies describing biological properties of plasma-modified PEEK surfaces mostly report increased cell adhesion. Argon plasma-treated PEEK contacted with fibroblast and epithelial cells showed an increase in cell adhesion [14]. Awaja et al. [47] modified PEEK using plasma immersion ion implantation using CH_4_/O_2_ mixture. The modified O-containing surfaces strongly increased MG63 adhesion and spreading. Plasma-treated PEEK was also proven to promote osteoblasts activity. In the case of ammonia-treated PEEK, in addition to an increase in osteoblasts adhesion, high cell activity (measured by detection of alkaline phosphatase and collagen) was also observed [15]. A similar increase in osteoblasts adhesion and activity was observed for PEEK treated with plasma in water vapor [48]. The biocompatibility of plasma-treated PEEK was also assessed using in vivo models. Oxygen-treated PEEK was implanted in sheep cancellous and cortical bone. The authors reported that no inflammation was observed. Also, the oxygen plasma surface modification improved osseointegration and implant stability [49].

It is known that the biological effectivity of the plasma treatment is depended on the process gases. In the case of osteoblasts, greater viability, and activity (measured by alkaline phosphatase activity) were demonstrated on surfaces treated with nitrogen compared to both surfaces treated with argon and argon + nitrogen mixture [16]. However, in this study, there was also a significant difference in surface topography between samples observed—the highest RMS value was measured for samples treated with nitrogen. Thus, in that case, the increase in cellular growth is probably the result of a synergistic effect of changes in chemical composition and topography.

However, some studies suggest that the cell adhesion enhancement on plasma-treated surfaces is caused by a change in the chemical composition rather than surface roughness. Fu et al. [17] showed a significant change in cell adhesion to plasma-modified PEEK, despite no change in surface roughness. Cell adhesion increased along with the following order: H-PEEK < O-PEEK < H/O-PEEK. Again, no significant change in roughness between those samples was observed.

In our research, we compared the adhesion of MG63 to the PEEK modified in the presence of nitrogen or oxygen. A similar study was carried out by Awaja, who compared MG63 adhesion to plasma-treated PEEK using a CH_4_/O_2_ mixture [47]. The modified O-containing surfaces strongly increased MG63 adhesion and spreading. Also, higher cell adhesion occurs at surfaces with higher oxygen and nitrogen content. The effect of both oxygen and nitrogen content on the number of surface-adhered cells was comparable—the correlation coefficient of cell adhesion was equal (0.74) in both types of surfaces.

**Figure 11:**
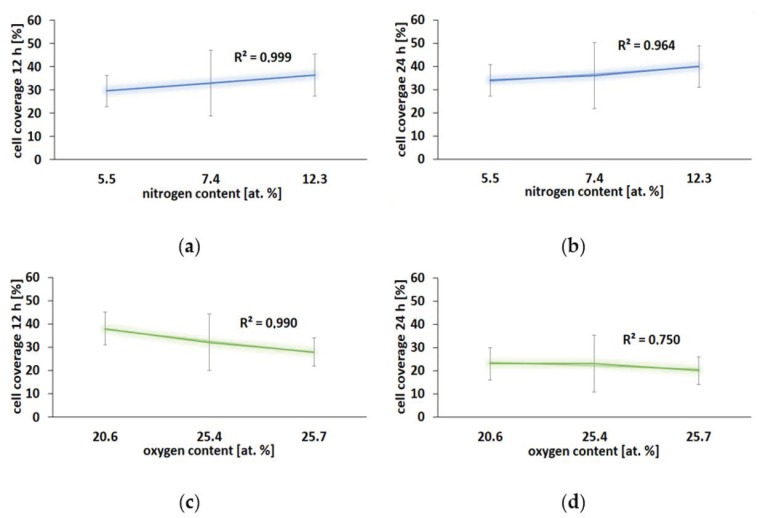
Cell adhesion to the analyzed surfaces as a function of nitrogen (**a**,**b**) and oxygen (**c**,**d**) content after 12 and 24 h of culture.

In our study, the correlation between nitrogen content and cell coverage was similar. The higher the nitrogen content in the surface, the higher cell coverage—correlation coefficient was >0.9 for both 12 h (Figure 11a) and 24 h (Figure 11b) of culture. However, in the case of surfaces modified in an oxygen atmosphere, we obtained an inverse relationship—the higher oxygen content the smaller cell coverage. This correlation was stronger for a shorter time of culturing (correlation coefficient = 0.990 for 12 h of culturing, Figure 11c) compared to the longer time of culturing (correlation coefficient = 0.750 for 24 h of culture, Figure 11d).

To summarize, in our study an increase in MG63 cell adhesion was achieved on all plasma-modified surfaces as compared to the control PEEK. The comparison of materials modified in the atmosphere of oxygen and nitrogen showed higher MG63 adhesion for materials modified in the nitrogen atmosphere. The effect was visible after 24 h of culture. In the case of materials with the highest nitrogen content (12.3 at.%), the value of cell coverage was significantly higher compared to materials modified in the oxygen atmosphere.

## 3. Materials and Methods

### 3.1. Modifications of PEEK Foils

**Figure 12:**
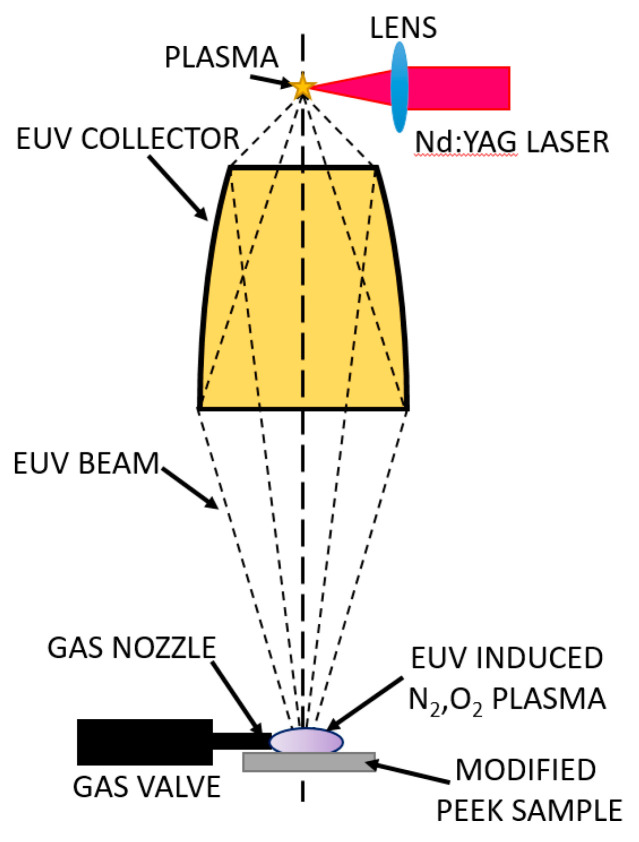
Scheme of the experimental arrangement for the modification of the PEEK surface with EUV radiation and nitrogen or oxygen plasma.

The amorphous PEEK foils (Goodfellow Cambridge Ltd., Huntingdon, UK) with a thickness of 0.075 mm were a subject of modification in the experiments presented here. The modifications were performed using a 10 Hz laser-plasma EUV source based on a double stream gas puff target irradiated with 4 ns Nd:YAG (λ = 1.06 µm) laser pulses with a pulse energy of 0.8 J (NL 303 HT, EXPLA, Vilnius, Lithuania). The target was created as a result of a pulsed injection of a xenon jet into a hollow stream of helium (Xe/He target) using an electromagnetic valve system equipped with a double nozzle setup. The laser beam focused on the stream of Xe created high-temperature plasma. The focusing conditions, as well as plasma parameters, were adjusted in order to obtain maximum intensity in the EUV spectral range. The EUV radiation was focused using a gold-plated grazing incidence ellipsoidal collector (RITE s.r.o., Kladno, Czech Republic). This collector allowed to efficiently collect, and focus radiation emitted from Xe plasma in the wavelength range of λ = 9–70 nm. The most intense emission attained was at a wavelength of 10 ± 1 nm. The EUV fluency in a focal plane of the collector reached about 60 mJ/cm^2^. The EUV beam was used for photoionization of nitrogen or oxygen gas injected into the region of the focal spot, perpendicularly to the optical axis of the irradiation system using an additional gas-puff valve. Nitrogen or oxygen gas density in the interaction region was controlled by adjustment of gas-puff valve opening time [50] and was 250 µs for each modification variant used in the experiment. The EUV induced, low-temperature nitrogen/(oxygen) plasma formed this way, together with an unabsorbed part of EUV radiation, was used for the surface modification of the PEEK foils—Figure 12. The samples were placed on the XYZ movable stage and located 3 mm from the EUV collector focal plane and irradiated with 100, 150, and 200 pulses at a 10 Hz repetition rate. The modified surface area of the PEEK samples was circular in shape with a diameter of ~1.9 mm. Such size of the modified surface was large enough for morphological examinations of PEEK foils (AFM, SEM). For chemical analysis (XPS), water contact angle, and biological examinations to be performed, the area of ~0.6 cm × 0.6 cm (7 spots in total) was modified.

### 3.2. Characterization of PEEK Surfaces

#### 3.2.1. Atomic Force Microscopy (AFM) and Scanning Electron Microscopy (SEM)

The effect of the modification of the PEEK surfaces in terms of morphology was analyzed using atomic force microscopy (AFM) (NT-MDT Spectrum Instruments, Moscow, Russia) and scanning electron microscopy (SEM) (Quanta FEG250, FEI, Boston, MA, USA). AFM allowed examining the surface topography, profile, and roughness of non-modified and modified polymer foils. The measurements were carried out at ambient conditions in a semi-contact mode using a golden silicon AFM probe (NSG10, NT-MDT Spectrum Instruments, Russia) featuring a pyramidal tip with a curvature radius of ~10 nm. The cantilever of the AFM probe was characterized by a force constant range of 3.1–37.6 N/m and a resonant frequency range of 140–390 Hz. The topographies collected were a scan size of 50 µm × 50 µm and with a resolution of 256 points per line. The cross-section analysis and roughness calculations were carried out using Image Analysis software provided by NT-MDT Spectrum Instruments.

For SEM observations, all samples studied were coated with a gold layer of 10 nm thickness. The gold layer was deposited from a gold target (99.999%) utilizing a sputter coater (Leica EM ACE600, Leica Microsystems, Wetzlar, Germany).

#### 3.2.2. X-ray Photoelectron Spectroscopy (XPS)

Chemical analysis of the surfaces of non-modified and modified PEEK samples with 100, 150, and 200 EUV pulses in the nitrogen and oxygen plasma presence was conducted using X-ray photoelectron spectroscopy. The spectrometer XPS (Prevac, Rogów, Poland) used was equipped with the analyzer SCIENTA R3000 (VG Scienta, Uppsala, Sweden) and an X-ray lamp with the Al K_α_ anode (Prevac, Poland). During the measurements, the pressure in the ultra-high vacuum analysis chamber of the XPS system was about 3 · 10^−9^ mbar.

The pristine PEEK sample was cleaned using ethyl alcohol, while the modified ones were examined without any cleaning treatment. High-resolution spectra in the narrow ranges of binding energy with a 40 meV step and pass energy of 100 meV for each band: C1s (294–282 eV), N1s (404.5–395.5 eV), and O1s (538–528 eV) were recorded. The peaks associated with C1s, N1s, and O1s bands were fitted using CasaXPS software. The background of Shirley type and Gaussian–Lorentzian (G–L) line shape (GL 50 for C1s, GL 60 for N1s, and GL 55 for O1s) were fitted for all these bands. All XPS spectra collected were shifted in such a way that the maximum of the C-C=C peak (refers to carbon ring in PEEK’s mer unit; carbon atom marked as 1—Figure 3) was at 284.8 eV.

#### 3.2.3. Water Contact Angle

In order to characterize the wettability of non-modified PEEK and PEEK modified with EUV radiation in the presence of oxygen and in the presence of nitrogen, the static water contact angle was measured with the sessile drop method at ambient temperature and humidity using distilled water (5 µL). The contact angle was measured automatically using the Kruss DSA 100 software (Hamburg, Germany). The measurement was performed in at least three randomly selected spots on the analyzed surface. Each variant of PEEK surface modification (plus non-modified one) was analyzed in triplicate (*n* = 9).

### 3.3. Cell Culture and Viability Tests

#### 3.3.1. Cell Culture

Human osteoblast-like MG63 cells (MG63, Lonza, Basel, Switzerland) were maintained and cultured in DMEM medium (Gibco/ThermoFisher Scientific, Waltham, MA, USA) supplemented with 10% FBS, 1% Pen-Strep, and 1% Glutamine (Gibco/ThermoFisher Scientific, Waltham, MA, USA). PEEK samples were cut to the form of discs (with a diameter of 5 mm) and sterilized with an antimicrobial solution containing penicillin (100 μg/mL), streptomycin (100 U/mL), and amphotericin B (0.25 μg/mL) for 1 h at 4 °C. After sterilization, materials were washed with PBS, placed in 48-well plates, and incubated with supplemented DMEM for 1 h at 37 °C. Next, cells were harvested, seeded on the material, and cultured in a humidified atmosphere with 5% CO_2_.

#### 3.3.2. Cell Viability

Cell viability was tested using the Alamar Blue assay (ThermoFisher Scientific, Waltham, MA, USA) according to the manufacturer’s protocol. Sterile non-modified and modified PEEK polymer samples were placed in 48-well plates, mounted with inserts, and incubated in 500 ul of supplemented DMEM medium for 1 h at 37 °C in a 5% CO_2_ humidified atmosphere. Following incubation, cells were seeded on the samples at the seeding density of 1 × 10^5^ cells/mL and cultured for a given time. Cells were also cultured in wells with no sample material (seeding density = 1 × 10^5^ cells/mL) and used as a control for viability calculation. After 24 h of culturing, the medium was removed from wells and 500 µL of Alamar Blue working solution (Alamar Blue reagent 10x diluted in fresh DMEM without phenol red) was added (500 µL/well). Samples were incubated for 4 h at 37 °C in a 5% CO_2_ humidified atmosphere (protected from light). Following incubation, 100 µL of Alamar Blue solution was transferred in triplicate to a 96-well plate (black) and fluorescence (Ex. = 550 nm, Em. = 590 nm) was measured. Samples were washed with PBS (3×, 5 min on a plate shaker), incubated with fresh DMEM, and used for further viability measurements. Viability results are presented as the ratio of positive control according to the equation:

Viability = FI sample_24_/FI control_24_

where:

FI sample–fluorescence intensity of the sample after 24 h of culturing,
FI control–fluorescence intensity of the control after 24 h of a culturing.

### 3.4. Cell Adhesion

After 12 and 24 h of culture, PEEK samples were analyzed with a confocal scanning microscope (CLSM, Zen 2, Zeiss, Oberkochen, Germany). At the end of the culture, samples were removed from the medium, washed with PBS, and fixed with 4% paraformaldehyde. The following staining procedure was applied: samples were rinsed 3 times with PBS, incubated for 4 min in 0.2% (*v*/*v*) Triton X-100, rinsed 3 times with PBS, incubated in 0.1% (*w*/*v*) Bovine Serum Albumin solution for 1 h, rinsed 3 times with PBS, incubated with AlexaFluor Phalloidin 488 for 30 min, rinsed 3 times with PBS, incubated with DAPI (4′,6-Diamidino-2-phenylindole dihydrochloride) for 5 min, and rinsed 3 times with PBS. Materials were glued to the cover glass with ProLong^®^ Gold Antifade Mountant (ThermoFisher Scientific, Waltham, MA, USA) and left until analysis.

Each variant of the modified PEEK surface was analyzed in triplicate, for every sample at least three randomly selected areas were depicted (*n* = 9). Images were analyzed using ImageJ (National Institutes of Health, Bethesda, Rockville, MD, USA), the percentage of the cell-coated area was calculated.

### 3.5. Statistical Analysis

Wettability and cell coverage were expressed as the means ± SD. Statistical significance of differences was analyzed using single-factor analysis of variance (ANOVA) with posthoc Tukey’s test (OriginPRO 2020b, OriginLab Corporation, Northampton, MA, USA). *p* < 0.05 were considered statistically significant.

## 4. Conclusions

In this paper, the influence of EUV irradiation and two low-temperature, EUV induced, oxygen and nitrogen plasmas on physical, chemical, and biological properties of PEEK surface was presented. It was found that the topography of the PEEK surfaces significantly changed due to modification. The nano- and micro- cone-like structures appeared on the surfaces, and the surface roughness increased compared to the unmodified PEEK (from 14.6 nm for pristine PEEK up to 253.3 nm for modified PEEK). EUV radiation and two types of plasma treatment led to strong changes in chemical composition, too, manifested with the appearance of new various functional groups (13 for PEEK samples modified in the presence of nitrogen and 10 for PEEK samples modified in the presence of oxygen), incorporation of nitrogen atoms up to ~12.3 at.% (when modified in the presence of nitrogen) and doubling the oxygen content up to ~25.7 at.% (when modified in the presence of oxygen), compared to non-modified PEEK foil. Moreover, the modification in the presence of oxygen slightly increased the wettability of the modified PEEK surfaces whereas the PEEK surfaces treated using nitrogen gas did not show significant differences. The influence of physico-chemical changes of the modified PEEK samples on the behavior of human osteoblast-like MG63 cells was evaluated. Cell viability performed after 24 h of culture revealed that all modified PEEK surfaces were not cytotoxic. Moreover, an increase in MG63 cell adhesion was achieved on all EUV and plasma-treated surfaces compared to the pristine PEEK. Comparison of PEEK polymers modified in the presence of oxygen and nitrogen shows higher MG63 adhesion for PEEK surfaces modified in the presence of nitrogen. The effect was visible after 24 h culture. As for PEEK surfaces with the highest nitrogen content (12.3 at.%), the value of cell coverage was significantly higher compared to PEEK surfaces modified in the oxygen atmosphere.

## Figures and Tables

**Table 1:** Binding energy range, FWHM range, and atomic concentration of different functional groups for pristine PEEK and modified PEEK (pristine PEEK peaks given in rows with blue background).

Symbol of the Peak	Chemical Group	Position [eV]	FWHM [eV]	PEEK [at.%]	PEEK -N_2_- 100 [at.%]	PEEK -N_2_- 150 [at.%]	PEEK -N_2_- 200 [at.%]	PEEK -O_2_- 100 [at.%]	PEEK -O_2_- 150 [at.%]	PEEK -O_2_- 200 [at.%]
C1	C-C=C	284.8	1.3	62.3	15.7	22.2	15.5	10.7	10.9	7.2
C2	C*-O-C*	286.4	1.6–1.7	19.9	11.1	7.4	4.9	14.9	8.9	6.3
C3	C=O	286.7	1.7–1.8	4.0	1.4	1.3	2.2	0.9	0.6	0.5
C4	C sp^2^	284.4	1.4	-	15.9	9.2	13.5			
C5	C*-COO	285.4–285.8	1.4–1.5	-	30.9	32.1	26.7	36.3	12.2	15.2
C6	C*-N	286.4–286.5	1.5–1.6	-	2.8	5.0	8.6			
C7	C*-OH	286.7–286.9	1.5–1.6					8.4	29.6	25.0
C8	O-C*-O	287.5–287.6	1.3–1.6	-	4.4	3.0	2.5	2.4	2.7	9.2
C9	O=C*-O	288.0–288.1	1.5–1.7					3.1	3.5	4.1
C10	N-C*=O	288.1-288.2	1.6–1.7	-	0.7	1.9	3.0			
C11	C*=O(OH)	288.9–289.2	1.4–1.7	-	1.5	1.3	1.2	1.2	2.5	2.3
C12	C*-OON	289.4–289.5	1.3–1.5	-	-	0.3	0.4			
C13	C*(=O)-O-C=O	290.2–290.3	1.5–1.6					1.4	3.2	3.8
C14	O-C*-O(=O)	291.0–291.3	1.4–1.5					0.2	0.5	0.8
N1	N*-C	399.3–399.4	2.1	-	2.8	5.0	8.6			
N2	N*-C=O	400.2–400.3	1.8	-	0.7	1.9	3.0			
N3	N*-x	400.9–401.1	2.1	-	2.0	0.5	0.7			
O1	O*=C	531.1	1.5	4.0	1.4	1.3	2.2	0.9	0.6	0.5
O2	C-O*-C	533.2	1.6	9.9	5.5	3.7	2.4	7.5	4.4	3.2
O3	C-O*H	531.9–532.1	1.4	-	1.5	1.3	1.2	1.7	0.7	0.7
O4	N-C=O*	532.2	1.5–1.6	-	0.7	1.9	3.0			
O5	OH (water)	534.2–535.5	1.6–1.8	-	1.1	1.0	0.6	0.5	5.6	7.6
O6	O=C-O*	534.2–534.3	1.8–1.9		-	-		10.0	14.1	13.7

**Table 2:** The atomic concentration of carbon, nitrogen, and oxygen for unmodified PEEK and modified PEEK.

Elements	PEEK [at.%]	PEEK-N_2_-100 [at.%]	PEEK-N_2_-150 [at.%]	PEEK-N_2_-200 [at.%]	PEEK-O_2_-100 [at.%]	PEEK-O_2_-150 [at.%]	PEEK-O_2_-200 [at.%]
C	86.2	84.4	83.7	78.5	79.5	74.6	74.4
N	-	5.5	7.4	12.3	-	-	-
O	13.9	10.2	9.2	9.4	20.6	25.4	25.7
